# Cyanobacterial Bloom Formation by Enhanced Ecological Adaptability and Competitive Advantage of *Microcystis*—Non-Negligible Role of Quorum Sensing

**DOI:** 10.3390/microorganisms12071489

**Published:** 2024-07-20

**Authors:** Ziqing Zhang, Jieming Li

**Affiliations:** 1 College of Resources and Environmental Sciences, China Agricultural University, Beijing 100193, China; zhangzqzgnydx@163.com; 2 Beijing Key Laboratory of Biodiversity and Organic Farming, China Agricultural University, Beijing 100193, China

**Keywords:** cyanobacterial bloom, colony formation, *Microcystis*, N-acyl-homoserine lactone, quorum sensing, quorum quenching

## Abstract

*Microcystis*-dominated cyanobacterial blooms (MCBs) frequently occur in freshwaters worldwide due to massive *Microcystis* colony formation and severely threaten human and ecosystem health. Quorum sensing (QS) is a direct cause of *Microcystis* colony formation that drives MCBs outbreak by regulating *Microcystis* population characteristics and behaviors. Many novel findings regarding the fundamental knowledge of the *Microcystis* QS phenomenon and the signaling molecules have been documented. However, little effort has been devoted to comprehensively summarizing and discussing the research progress and exploration directions of QS signaling molecules-mediated QS system in *Microcystis*. This review summarizes the action process of N-acyl homoserine lactones (AHLs) as major signaling molecules in *Microcystis* and discusses the detailed roles of AHL-mediated QS system in cellular morphology, physiological adaptability, and cell aggregation for colony formation to strengthen ecological adaptability and competitive advantage of *Microcystis.* The research progress on QS mechanisms in *Microcystis* are also summarized. Compared to other QS systems, the LuxI/LuxR-type QS system is more likely to be found in *Microcystis*. Also, we introduce quorum quenching (QQ), a QS-blocking process in *Microcystis*, to emphasize its potential as QS inhibitors in MCBs control. Finally, in response to the research deficiencies and gaps in *Microcystis* QS, we propose several future research directions in this field. This review deepens the understanding on *Microcystis* QS knowledge and provide theoretical guidance in developing strategies to monitor, control, and harness MCBs.

## 1. Introduction

Cyanobacterium, an ancient unicellular photosynthetic prokaryote, is crucial in the emergence of high-level aerobic organisms [1]. With global warming and the rising eutrophication of water bodies, cyanobacterial blooms frequently occur worldwide via massive cell proliferation [2,3]. *Microcystis* is the most common genus that dominates cyanobacterial blooms in freshwaters and consists of two ecotypes, microcystin-producing (MC^+^) and non-microcystin-producing (MC^−^) *Microcystis* [3,4], where microcystins (MCs) are widespread cyanotoxins that endanger eco-safety [5]. Over the past few decades, outbreaks of MC^+^ *Microcystis*-dominated cyanobacterial blooms (MCBs) in freshwaters have endangered ecological and human health and aroused public concern [6,7,8]. The development and depletion of MCBs involve following processes: *Microcystis* cell aggregation, colony formation, and colony disaggregation [9]. *Microcystis* cell aggregation into colonies enhances their buoyancy to allow for rapid flotation nearby the freshwater surface, so their colony formation contributes to MCBs outbreak/maintenance and *Microcystis* dominance/prevalence in cyanobacterial blooms [10,11,12]. From the colony formation view, many researchers have explored the cause of MCBs outbreak [13,14,15,16,17], while the regulatory mechanisms of *Microcystis* colony formation deserve further attention. Although most studies suggested that the production and secretion of MCs and extracellular polymers (EPSs), as well as other cellular traits, of *Microcystis* lead to *Microcystis* cell aggregation and colony formation, it is noteworthy that QS is directly responsible for *Microcystis* colony formation. Thus, QS as a direct driving force for *Microcystis* colony formation is non-negligible.

QS is a density-dependent phenomenon that regulates intercellular communication between and within species. The QS system is prevalent in bacterial kingdom, with the majority reported for Gram-negative (G^−^) bacteria. The bacteria with QS system can secrete signaling molecules called autoinducers (AIs), where the secretion increases with rising cell density to regulate bacterial population characteristics and behaviors [18]. When diffusible signaling molecules accumulate to the required threshold in an extracellular environment, the bacterial population exhibits obvious changes in phenotypic and behavioral traits, involving cell aggregation, swarming motility, bioluminescence, biofilm formation, colonization, sporulation, cytotoxins/antibiotics syntheses, and virulence factor production [19,20,21,22]. Generally, QS exerts its effect via three stages, as follows: signaling molecule-production/secretion by cells in a population, signaling molecule-sensing by other cells within or outside the population, and signaling molecule-binding by the receptor protein. The QS phenomenon has recently been discovered in *Microcystis* population [23]. The regulatory roles and processes of QS in the *Microcystis* population behaviors and in MCBs outbreak/maintenance are becoming a research hotspot and are well-documented currently. However, little effort has been dedicated to comprehensively summarizing and discussing the research progress and exploration directions of *Microcystis* QS.

The literature search is predominantly executed within the databases of Web of Science, PubMed, Scopus, and China National Knowledge Infrastructure by employing a keyword strategy encompassing combinations, including (‘*Microcystis*’ OR ‘*Microcystis aeruginosa*’ OR ‘*M*. *aeruginosa*’ OR ‘Algae’ OR ‘Cyanobacteria’) in conjunction with (‘Quorum sensing’ OR ‘Quorum quenching’), through which we procured relevant studies that span the publication period from 1990 to 2024. Based on these studies, this paper provides an updated comprehensive summary and review for current research progresses of *Microcystis* QS, aiming to facilitate an integrative understanding on QS-regulated MCBs outbreak mechanisms. Specifically, the discovery processes of the QS phenomenon and QS signaling molecules in *Microcystis* are first described. The detailed roles of QS signaling molecules secreted by *Microcystis* itself or those exogenously originated in *Microcystis* cell aggregation and colony formation are reviewed and discussed from several aspects of cell growth, cellular morphology, physiological adaptability (i.e., MCs and EPSs production/release), and nutrition and energy metabolism activities. To grasp QS mechanisms, genetic information and action process, including signal syntheses, sensing, and binding of *Microcystis*, are summarized. Various exogenous signal analogs as QS inhibitors and QS-blocking process in *Microcystis* through degrading QS signals, disrupting QS signals syntheses and signals receptor-binding are introduced, with emphasis on the potential of QS inhibitors in MCBs control. Ultimately, we propose several future research directions in the field of *Microcystis* QS, with the purpose of deepening the understanding on the knowledge in this field and developing strategies to monitor, control, and harness MCBs in the context of climate change.

## 2. QS Discovery and Signaling Molecule Recognition in *Microcystis*

### 2.1. Cell Density-Dependent Regulatory Behaviors in Microcystis

QS regulates bacterial population characteristics via signaling molecule-mediated intercellular communication, where an individual cell can perceive cell density by sensing the concentration of signaling molecules diffused in the population. When cell density reaches the responding threshold, the cells adjust functional gene expression to alter phenotypic traits. Thus, QS eventually regulates population characteristics in such a manner that cannot be achieved by a single cell [24,25]. *Microcystis*, as G^−^ bacterium-like prokaryote, has a similar cellular structure to G^−^ bacteria. To explore fundamental mechanisms of MCBs outbreak for better MCBs control/management, many studies aimed to discover QS phenomenon in *Microcystis*.

Using a semi-continuous culture experiment, Pereira et al. [23] observed that different cell densities led to obviously various metabolites in *Microcystis* and proposed the presence of QS phenomenon that causes the production of different metabolites in *Microcystis*. Wood et al. [26,27] found a significant positive correlation between MCs amount and *Microcystis* cell density, where MCs amount produced by each cell increases with rising cell density, suggesting that *Microcystis* cell density could drive some physiological processes. Wang et al. [28] and Xie [29] experimentally confirmed that the expression level of MCs-synthesizing genes and the enhanced MCs concentration were positively proportional to *Microcystis* cell density, which further indicated that *Microcystis* cells could adjust their physiological metabolism and cell aggregation (mediated by MCs) by perceiving cell density. Yet, the above studies could not give a definite answer to the question of whether QS exists in *Microcystis* until the signaling molecules and their regulatory manner were identified in *Microcystis*.

### 2.2. Discovery and Recognition of QS Signaling Molecules in Microcystis

Bacterial QS signaling molecules are classified into four major categories: N-acyl-homoserine lactones (AHLs), autoinducer peptides (AIPs), autoinducer-2 (AI-2) and autoinducer-3 (AI-3) [30,31,32]. Typically, G^−^ bacteria mainly possess AHL-mediated QS system that secretes AHLs as signaling molecules for intraspecific communication. AHL-mediated QS system has multiple signal–receptor gene homologs, including LuxI/LuxR, LasI/LasR, RhlI/RhlR, AfeI/AfeR, BtaI/BtaR, and TofI/TofR [33]. AIPs are major QS signal molecules in Gram-positive (G^+^) bacteria for their intraspecific communication, while AI-2 (encoded by *luxS* gene) is an interspecific communication signal secreted by both G^−^ and G^+^ bacteria [34]. AI-3 is a metabolite of previously unknown structure involving pathogenesis of *Escherichia coli*, which drives bacteria/host inter-kingdom communication [35,36].

**Figure 1:**
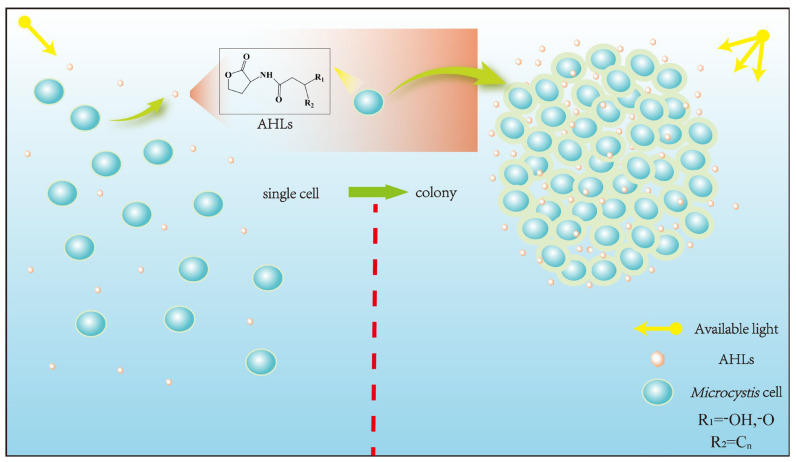
The process of *Microcystis* colony formation mediated by AHLs. The AHLs secreted by *Microcystis* increased with rising *Microcystis* cell density. In the process of cell proliferation, single *Microcystis* cell can aggregate to form the *Microcystis* colony under the influence of AHLs. The general structure of the N-acyl-homoserine lactone is shown, where R_1_ = -OH, -O and R_2_ = C_n_.

*Microcystis* possesses a similar cellular structure to G^−^ bacteria; thus, most studies speculated that the QS system of *Microcystis* might be similar to that of G^−^ bacteria, where AHLs acted as signaling molecules to regulate *Microcystis* population characteristics [18]. The molecular structure of AHLs consists of two parts, a homoserine lactone (HSL) ring and a variable amide side chain, so the diversity of AHLs structures is caused by differences in side chain length, substituent group, and substituent position [37,38,39]. AHLs comprise short-chain (C4-HSL~C8-HSL) and long-chain (C10-HSL~C18-HSL) homologs according to side chain length, while -OH and -O groups, as common substituent groups, often replace the H atom at C3 position of AHLs (Figure 1). Sharif et al. [40] demonstrated that the cyanobacterium *Gloeothece* produces C8-HSL in axenic culture. Over the recent years, a growing number of studies have successfully extracted AHLs from axenic culture of *Microcystis* and confirmed that AHLs could be synthesized and secreted by *Microcystis*. For the first time, Zhai et al. [41] used liquid chromatography–mass spectrometry and bioreporter assay to evidence that *Microcystis* can produce AHLs in axenic culture. Meanwhile, researchers revealed that AHLs concentration changed with dependence on *Microcystis* cell density, where AHLs concentration peaked as *Microcystis* cell density reached the required threshold [29,41]. It was also found that an obvious accumulation of AHLs occurred only when cell density threshold reached, which accorded with the regulatory manner of many QS systems [40]. The above studies proved that *Microcystis* could depend on cell density to adjust AHLs production and secretion. Namely, *Microcystis* can produce AHLs as signaling molecules based on cell density, which has a potential to induce a series of physiological/behavioral changes at the concentration threshold of AHLs. The AHLs homologs produced by different *Microcystis* strains are summarized in Table 1. Table 1 presents information about previous studies that successfully extracted the molecules of AHLs from *Microcystis*, including media, molecular formula, as well as *Microcystis* strains.

## 3. AHLs’ Roles in Regulating Physiology and Colony Formation of *Microcystis*

### 3.1. Regulatory Effects of Endogenous AHLs

The roles of endogenous AHLs produced by *Microcystis* have been widely explored. Many studies extracted AHLs from *Microcystis* cells and added such endogenous AHLs into *Microcystis* culture at the growth stage to observe physiological and population changes in *Microcystis* [28,41,42,43,44,45]. Endogenous AHLs were found to affect *Microcystis* in many aspects, including cellular morphology, physiological adaptability, nutrition and energy metabolism activities, and triggering cell aggregation for colony formation.

#### 3.1.1. Cellular Morphology

The secreted AHLs can deform cell walls and promote the formation of more gas vesicles in *Microcystis* with increasing cell density. These columnar-shaped gas vesicles regulate cell buoyancy, which enables *Microcystis* to occupy the surface layer of the water body that favors its competitive advantage maintenance in aquatic ecosystem [46]. For instance, Xu et al. [43] identified novel long-chain AHLs from *Microcystis* and demonstrated a significant correlation between AHLs concentration and cell density. By adding the AHLs into culture, the authors also confirmed that the AHLs enhanced *Microcystis* cell buoyancy and the expression of vesicle-related genes. This result evidenced that *Microcystis* could secrete AHLs to enhance cell buoyancy via vesicle formation, which allows *Microcystis* to occupy the water surface layer to become an advantageous competitor [29].

#### 3.1.2. Cellular Physiological Adaptability

Endogenous AHLs can regulate and modify the production of MCs and EPSs. As widely reported, MCs may strengthen the ecological fitness of *Microcystis* by resisting biochemical stressors (e.g., hydrogen peroxide, metal ions, predators) [47,48], raising the adaptability to high-radiation and oxidation conditions [48,49], enhancing competitiveness over its MC^−^ counterparts [50], promoting large-size colony formation [51,52,53], and helping *Microcystis* overwintering and recovery from cold environment [54,55]. EPSs also improve *Microcystis* resistance to stresses caused by many factors, such as grazing pressure by protozoa and allelopathic pressure by anti-cyanobacterial allelochemicals [56,57,58]. Thus, *Microcystis* can improve ecological adaptability and stress-resistance by adjusting MCs and EPSs contents, which could be driven by AHLs. Wang et al. [28] observed that the concentration of MC-LR (a common homolog of MCs) increased with *Microcystis* cell density in growth culture but remained stable when cell density was kept constant by adding medium into the culture, indicating a close correlation between the MCs production and cell density of *Microcystis*. Meanwhile, the authors detected a similar trend in AHLs concentration, with AHLs being detectable even at low cell density where MCs were undetectable. This phenomenon reflected that the MCs increase was caused by AHLs. Additionally, Xu et al. [43] observed the promotive effects of AHLs on MC-LR secretion and MCs-synthesizing gene expression by using qPCR analysis.

#### 3.1.3. Nutrition and Energy Metabolism Activities

Some AHLs homologs can activate relative enzymes to improve carbon and nitrogen metabolism efficacy in *Microcystis* and can also adjust the synthesis and secretion of various metabolites by affecting the gene expression and enzyme activity of multiple pathways. For instance, Yan et al. [42] identified 3-OH-C4-HSL (a homolog of AHLs) as the QS signaling molecule of *Microcystis* and verified that the addition of 3-OH-C4-HSL could up-regulate QS-related genes *Dpp* and *Sec*, and the expression of genes related to NADH dehydrogenase, succinate dehydrogenase, cytochrome c oxidase was also up-regulated to promote the expression of all ATP-synthesizing genes. Consequently, carbon and energy metabolisms were promoted in *Microcystis*. This suggested that the homolog of AHLs acted as the trigger to initiate a series of downstream metabolisms. Xu et al. [59] found the up-regulated expression of photosynthesis-related genes (e.g., *apcABF*, *petE*, *psaBFK*, *psbUV*) promoted nitrogen metabolism and ribosomal metabolism and increased the content of chlorophyll by the action of AHLs extract.

#### 3.1.4. Colony Formation

AHLs influence *Microcystis* cell aggregation to promote colony formation, which involve EPSs concentration and composition controlled by AHLs [60]. As reviewed above, AHLs also affect cellular morphology, physiological adaptability, and metabolism activities of nutrition and energy to expand the advantage of *Microcystis* in waters, thereby jointly affecting its cell growth and colony formation. Zhai et al. [41] found the existence of a special AHLs molecule named (E)-7-hydroxy-5-oxo-N-(2oxotetrahydrofuran-3-yl) oct-2-enamide in the pure culture of *Microcystis aeruginosa* PCC-7820, and such AHLs promoted cell aggregation. The sticky EPSs possess various functional groups (e.g., -OH, C-O) that strongly bind with Ca^2+^ and Mg^2+^ in waters, which assist *Microcystis* cells bind together to form bio-aggregates alike to flocculent sludge, bioparticle, and biofilm [61,62]. Zhai et al. [41] revealed that AHLs extracted from *Microcystis* promoted biofilm formation to induce cell aggregation. Xu et al. [63] discovered a decreased aggregating ability of *Microcystis* after cellular EPSs were extracted, and such a decrease in *Microcystis* aggregation was more obvious in field samples than in lab-culture. Noteworthy, most previous studies ascribed *Microcystis* cell aggregation to the stickiness of extracellular polysaccharides (ex-poly) in EPSs. However, more recent studies proposed the key function of extracellular proteins (ex-pro) of EPSs in promoting cell aggregation and colony formation. For instance, Xu et al. [43] found that AHLs extracted from *Microcystis* did not obviously promote EPSs syntheses and secretion but increased ex-pro content in EPSs to enhance Zeta potential and hydrophobicity, thus promoting *Microcystis* cell aggregation. Using bioinformatics and comparative genomics analyses, Qiu [18] found the existence of genes encoding PEP-CTERM domain proteins and suggested that these genetically controlled proteins secreted on the cell surface may form complex polymers with ex-poly through glycosylation process to induce cell aggregation and colony formation.

**Figure 2:**
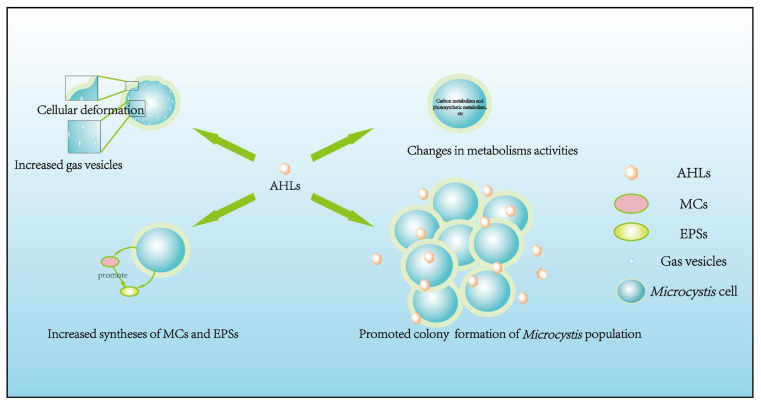
Regulatory effects of endogenous AHLs on various aspects of *Microcystis*. These aspects include cellular morphology, physiological adaptability, nutrition and energy metabolism activities, and colony formation.

The regulatory process of AHL-mediated QS for *Microcystis* colony formation is shown in Figure 1. Concretely, AHLs concentration can increase with rising cell density. When cell density reaches a specific threshold, AHLs concentration culminates in an aqueous phase to promote *Microcystis* cell aggregation, survival, and competitive advantages by improving cellular morphology, physiological adaptability, nutrition and energy metabolism activities, thus promoting colony formation for MCBs occurrence (Figure 2).

### 3.2. Regulatory Effects of Exogenous AHLs

Besides endogenous AHLs produced by *Microcystis*, researchers also conducted extensive studies to explore the effects of exogenous AHLs on *Microcystis*. The premise of exploring exogenous AHLs’ function is that these AHLs produced by other organisms can affect *Microcystis*. Yan et al. [42] found that the addition of 3-OH-C4-HSL extracted from bacterial sludge could promote microalgal growth, like the function of endogenous AHLs, whereas Xue et al. [64] found that exogenous AHLs addition decreased *Microcystis* growth rate. Based on existing studies, exogenous AHLs affect *Microcystis* in several of the same aspects as endogenous AHLs. This means that both endogenous and exogenous AHLs influence the same four aspects of *Microcystis*, including cellular morphology, physiological adaptability, nutrition and energy metabolism activities, and cell aggregation for colony formation (Figure 2).

By adding exogenous AHLs into *Microcystis* culture, Xie [29] showed that exogenous AHLs could change cell wall shape, and the size of gas vesicles increased under the action of C4-HSL and C8-HSL and also found that some exogenous AHLs promoted *Microcystis* cell growth. Among them, the promotive effect of 3-OH-C4-HSL is most obvious. However, exogenous C8-HSL had no promotive effect on *Microcystis* cell growth and even decreased the expression level of genes related to ATPase and hydrolyase activity [29]. In the aspect of physiological adaptability, the EPSs and MCs content of *Microcystis* can be regulated and controlled by exogenous AHLs. Exogenous AHLs were observed to promote the EPSs secretion of *Microcystis*, and the exogenous AHLs with growth-promoting effect could decrease MCs secretion but increase MCs syntheses, while the ones without growth-promoting effect caused high MCs content. This suggested that the exogenous AHLs may pose opposite effects on EPSs and MCs secretion [29]. In the aspect of nutrition and energy metabolism activities, a part of exogenous AHLs can change relative gene expression and enzyme activities to regulate metabolism processes such as carbon and nitrogen metabolism in *Microcystis* [29,65,66]. C6-HSL can increase carbon sequestration efficiency to promote *Microcystis* growth [64]. Under joint action of the above three aspects, *Microcystis* cell growth and aggregation for colony formation also changed. For instance, N-octanoyl-L-homoserine lactone (C8-HSL), N-(3-oxooctanoyl)-L-homoserine lactone (3-oxo-C8-HSL), and N-butyryl-DL-homoserine lactone (C4-HSL) can promote the formation of biofilm-like membrane on *Microcystis* and, thus, significantly strengthen colony formation [41,67].

Notably, there is a close correlation between MCs syntheses/secretion and EPSs composition of *Microcystis* [51]. MCs can up-regulate ex-poly synthesis-related genes such as *capD*, *csaB*, *tagH*, and *epsL* to significantly increase ex-poly content in EPSs [52,68,69]. As a major component of EPSs, the tightly bound ex-poly can facilitate capsule formation around *Microcystis* cells to increase cell surface viscosity and promote colony formation [18,69,70,71,72]. This provides further explanation for the positive correlation between MCs content and colony size. Based on the above, AHLs not only act on MCs and EPSs secretion alone but also regulate the linkage of MCs and EPSs.

Diverse AHLs homologs with different molecular structures can exert vastly distinct functions in the QS regulation pathway of G^−^ bacteria [37,38,39]. Likewise, different AHLs homologs also exert distinct effects on *Microcystis* [29,67]. As revealed by Lamas-Samanamud et al. [44] and Xie [29], the AHLs with phenyl groups, oxo groups, ether groups, and bromide substituents decreased MCs content in the aqueous phase [44], and the AHLs differing in hydrophilicity and side chain length seemed to pose different influences on MCs syntheses and the secretion of *Microcystis* [29]. Most studies focused on how cell growth, cellular morphology, cell aggregation for colony formation, chlorophyll a content, and the photosynthesis of *Microcystis* were affected by adding exogenous AHLs. The effects of different added exogenous AHLs on various aspects of *Microcystis* are summarized in Table 2. The addition of exogenous AHLs could mimic a naturally algae-bacteria co-existent circumstance where *Microcystis* is affected by AHLs secreted by other algae and/or bacteria. Owing to different exogenous AHLs used for addition experiments, the observed effects were always divergent (e.g., promotive or inhibitory effect) among experiments. Applying exogenous AHLs with an inhibitory effect on *Microcystis* growth provides a new option for controlling and restricting MCBs outbreak.

## 4. Genetic Information of AHL-Mediated QS in *Microcystis*

The AHL-mediated QS system widely prevalent in G^−^ bacteria is termed the LuxI/LuxR-type QS system, which includes AHLs synthase (termed ‘LuxI protein’) and AHLs receptor (termed ‘LuxR protein’), with AHLs as signaling molecules [38,74]. In such a system, the *luxI* gene encodes the AHLs synthetase, while the *luxR* gene encodes the AHLs receptor to sense AHLs and become transcription activators for other genes (e.g., *luxI*, *luxA*, *luxB*, *luxC*, *luxD*, *luxE*), among which *luxA*-*E* participate in regulating specific behaviors caused by QS [31,75,76,77,78,79,80,81]. AHLs synthesized in cells can be diffused to surroundings via simple diffusion and/or transport by specific transporter [24]. With rising cell density, AHLs gradually accumulate in surroundings. Only when AHLs concentration accumulates up to a specific threshold can AHLs bind with LuxR protein to form signal–receptor complex dimers/multimers to activate downstream gene expression and induce corresponding behavioral changes in G^−^ bacteria [72]. The AHLs concentration threshold required for activating specific gene expression is highly specific. AHLs act as a trigger to activate differential functional genes in sequence when each specific threshold is reached [80,82].

Because *Microcystis* shares a similar cellular structure and similar category of QS signaling molecules to G^−^ bacteria, the following question is asked: ‘does any similarity present in genetic mechanisms of QS between *Microcystis* and G^−^ bacteria?’ Xie [29] conducted genetic-level research for QS in *Microcystis* and proposed that AHLs initiated QS by affecting the expression of QS-related genes, such as BH695_RS06140, but the authors did not identify the gene function. Chen [66] identified the *slr2100* and *slr1259* genes that are homologous to the *luxI* and a*iiA* genes (encoding AHL–lytic enzyme), respectively, in cyanobacterium *Synechocystis* sp. PCC6803 and found that *slr2100* and *slr1259* gene expression was detected in *Microcystis* at different growth stages. This implied that *Microcystis* could express homologous proteins to LuxI and AiiA for AHLs syntheses and degradation, respectively, which caused the self-regulation of the AHLs level. Chen [66] proposed that the QS system in *Microcystis* was similar to the AHL-mediated QS system of G^−^ bacteria (i.e., LasI/LasR-type QS system in *Pseudomonas aeruginosa*). Interestingly, Lamas-Samanamud et al. [81] found *luxS* gene expression in *Microcystis aeruginosa* PCC7806. The *luxS* gene encodes the crucial enzyme for synthesizing AI-2, which is the QS signaling molecule for another QS system [24,30,31]. Despite this finding, AI-2 as a QS signal has not yet been identified in *Microcystis* until now. Hence, it is unclear whether there is an AI-2-mediated QS system in *Microcystis* and whether the *luxS* gene in *Microcystis* functions the same as in bacteria that encode AI-2 synthetase.

**Figure 3:**
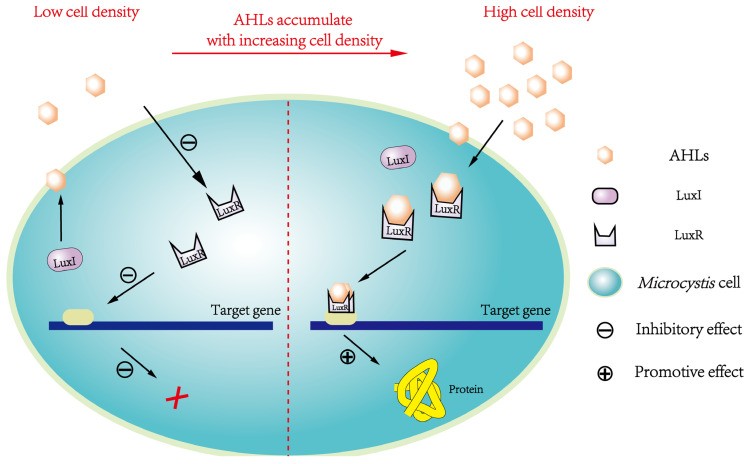
Schematic diagram of the LuxI/LuxR-type QS system mediated by AHLs that may present in *Microcystis*, where LuxI controls the AHLs syntheses, and LuxR is AHLs receptor for AHL-sensing. When the concentration of AHLs reaches a specific level, AHLs can bind to receptor and form the complex of LuxR and AHLs to activate the expression of related genes.

At present, the enzymes and signaling molecules have not been detected simultaneously in the above two possible QS systems. However, the LuxI/LuxR-type QS system is more similar to the QS system in *Microcystis*, based on the evidence of *slr2100* and *slr1259* gene expression found in *Microcystis* [66]. This system model suggests that (i) when *Microcystis* cell density is low, AHLs concentration remains at a low level, so AHLs cannot be sensed and bound by AHLs receptor, thus failing to activate various gene expression; (ii) when *Microcystis* cell density is high, AHLs concentration increases to a high level, so AHLs can bind with AHLs receptor to form signal–receptor complexes, which in turn activate AHLs syntheses and induces other specific gene expression to produce functional proteins (Figure 3). However, substantial conclusive evidence for LasI/LasR-type or LuxI/LuxR-type QS system presence in *Microcystis* is still insufficient, and genetic information for AHL-mediated QS in *Microcystis* remains largely lacking, which deserves urgent exploration.

## 5. Quorum Quenching (QQ) of *Microcystis* and Application Implication

QQ is a phenomenon that negatively disrupts microbial colony formation and population behavior by interfering with any step of the QS process, such as inhibiting AHLs syntheses, promoting AHLs degradation, and disrupting AHLs binding to receptor [29]. In G^−^ bacteria, several substances have an inhibitory effect on AHLs syntheses, such as the structural analogs of intermediates during the AHLs syntheses process, purine nucleotide, the derivative of HSL, and the homologs and analogs of some antibiotics [83]. Researchers have found some of these substances disrupt AHLs syntheses process by competing with intermediates [84]. For instance, as the AHLs synthase, LuxI can catalyze the formation of an amide bond between S-adenosylmethionine (SAM, an essential intermediate) and the acyl carrier protein for AHLs syntheses. The structural analogs of SAM (e.g., S-adenosylhomocysteine, S-adenosylcysteine, and sinefungin) have an inhibitory effect on AHLs syntheses to cause QQ. Parsek et al. [84] identified that such inhibitory function might be caused by the competition between the SAM analogs and SAM.

**Figure 4:**
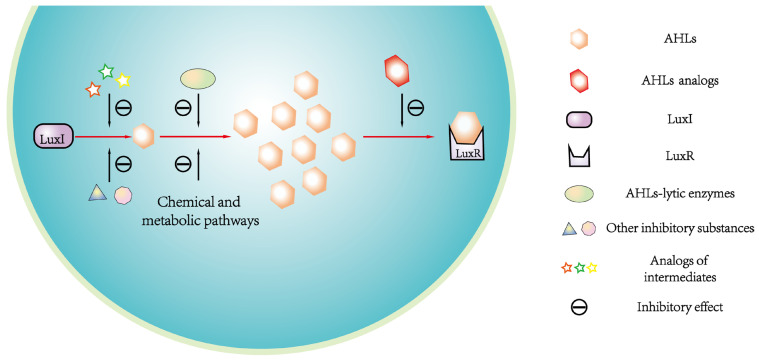
Schematic diagram of QQ mechanisms against the LuxI/LuxR-type QS system in *Microcystis*. The QS can be quenched by disrupting the pathways of AHLs syntheses, AHLs accumulation, and AHLs binding to the receptor.

Additionally, AHLs can be degraded in vivo through biochemical metabolisms and specific enzymes, including AHL–acylase, AHL–lactonase, and oxidoreductases, and synthetic AHLs structural analogs compete with corresponding AHLs signals for the binding sites of AHLs receptor; thus, AHLs binding to receptor can be prevented [38,71,82]. The expression of the *sll1392* gene that is homologous to the *luxR* gene was suppressed, but the *slr1259* gene was stimulated after adding exogenous AHLs structural analogs (i.e., ɑ-amino-γ-butyrolactone hydrobromide) into *Synechocystis* culture [66]. Such a gene expression response to exogenous AHLs’ structural analog is similar to the corresponding response manner of the AHL-mediated QS system in G^−^ bacteria [66]. These suggested that cyanobacterial QS could be quenched by promoting AHLs degradation and disrupting AHLs binding to receptor, and the QQ mechanisms in cyanobacteria might be similar to those in G^−^ bacteria [66]. Romero et al. [71] indicated that an acylase named AiiC in cyanobacterium *Anabaena* sp. PCC 7120 might be responsible for the self-regulation of AHLs levels, and recombinant AiiC could degrade a series of AHLs molecules, thus causing QQ against QS signals. Although the homologous gene to *aiiC* was not identified in *Microcystis*, the *aiiA* homologous gene (*slr1259*) encoding AHL–lytic enzyme could be expressed in *Microcystis*, implying that AHLs degradation was mediated by different genes to cause QQ in different cyanobacteria. Based on the above, a schematic diagram for QQ mechanisms in *Microcystis* is summarized in Figure 4.

Consequently, the QS of *Microcystis* is not only regulated by AHLs signals that are secreted by its own cells but also negatively impacted by those secreted by other organisms such as bacteria. The principle of QQ provides a new strategy to develop eco-benign algicidal methods to control toxigenic MCBs caused by the excessive proliferation and colony formation of *Microcystis*. Exogenous AHLs produced by other algae and bacteria may impact *Microcystis* growth in algae–bacteria co-existence environments. For instance, Yang et al. [58] reported that some bacteria secreted α-amino-γ-butyrolactone hydrobromate (acyl-HSLs) to impact *Microcystis* growth, and Romero et al. [71] found that chlorophyta greatly disrupted cyanobacterial QS through QQ effect in their co-existence system. Based on this, researchers could control MCBs by introducing QQ signal-secreting microorganisms. To achieve QQ, many studies also applied exogenous AHLs and/or AHLs structural analogs as the QS inhibitors against *Microcystis* cell proliferation [65]. For instance, *Microcystis aeruginosa* and *Synechocystis* sp. growth were inhibited by the addition of acyl–HSLs [73]. The addition of 3-oxo-C10-HSL and C10-HSL caused the obvious inhibition of cell growth and colony formation of *Microcystis* [67].

Researchers also revealed that a variety of plant-originated and synthetic organic compounds could serve as QS inhibitors, such as vanillin extracted from vanilla beans and a heterocyclic oxygenic compound named furan [29,42]. Zhai et al. [41] found that one of the furanone, dihydro-3-amino-2-(3H)-furanone (FN), and *Microcystis*’s QS signaling molecules could bind to the same site on the receptor. Thus, FN could compete with QS signals of *Microcystis* for receptor, indicating that FN acted as a QS inhibitor by blocking receptor-binding. Further research reported that FN could bind to two residues, such as Asn164 (A) and His167 (A) of the AHLs receptor (i.e., LuxR), to form hydrogen bonds. The hydrophobic interactions between FN and binding pockets formed by residues Gln137(A), Val140-(A), Ala163(A), Asn164(A), and His167(A) also enabled FN to exhibit a more competitive advantage in receptor-binding than QS signals [42]. Such an advantage effectively inhibited the carbon assimilation and energy metabolism of *Microcystis* to control its cell density.

## 6. Future Perspectives

Compared to G^−^ bacteria, the research on the QS and QQ of *Microcystis* is relatively insufficient, especially in aspects of genetic mechanisms, so the information on QS-related and QQ-related genes and associated functions should be further screened and probed in *Microcystis*. Thus, bioinformatics (e.g., sequence blast and alignment) could be used to clarify whether *Microcystis* possesses QS-related and QQ-related genes homologous to those in *Anabaena* and other G^−^ bacteria. By screening new target genes, we can unveil the gene functions and track the action pathways of QS and QQ in *Microcystis* using gene knockout and isotopic tracer techniques for better understanding QS and QQ mechanisms of *Microcystis*, which will lay a solid theoretical basis for toxigenic MCBs control.

Owing to climate change and eutrophication, MCBs intensity and frequency may continuously increase [85], so the interaction of *Microcystis*’s AHLs and other microorganisms should be elucidated to facilitate understanding of the complex roles of *Microcystis*’s AHLs in aquatic ecosystems. It is also essential to develop an efficient and specific AHL-detection technology as technical support for harnessing MCBs by promptly gaining QS signal levels and AHLs species distributions. Meanwhile, how environmental factors (e.g., light, temperature, nutrient salt levels) affect aqueous AHLs species distributions and the AHLs’ effects should be considered, which is helpful to predict the AHL-mediated outbreak of MCBs as environment factors change in the context of climate change. Based on this review, we suggest following research directions that are crucial for advancing knowledge and understanding in this field and developing strategies to monitor, control, and harness MCBs, which deserve further attention and comprehensive research:

(i) Elucidating the genetic mechanisms for QS and QQ of *Microcystis*, especially clarifying related gene functions and action pathways, using modern molecular biotechnologies and bioinformatics;
(ii) Further identifying AHLs species produced by *Microcystis* and exploring their effects on the growth and physiology of many other bloom-forming algae;
(iii) Exploring other QS-signaling molecules besides AHLs in *Microcystis* and whether some AHLs species produced by *Microcystis* can inhibit *Microcystis*’s QS, then comparing the molecular structure difference between QS-inducing and QS-inhibiting AHLs for *Microcystis*;
(iv) Surveying the effects of diverse environmental factors on aqueous AHLs species distributions and AHLs’ effects.

## 7. Concluding Remarks

*Microcystis* can secrete AHLs as QS-signaling molecules to regulate its population characteristics and behaviors. As previously proposed, the diffused concentration of endogenous AHLs increases with rising cell density until a specific threshold is reached for AHLs receptor-binding to form a signal–receptor complex, which can further activate functional gene expression. Consequently, an AHL-mediated QS system regulates physiological/behavioral changes in the *Microcystis* population. The QS system promotes the morphological and physiological adaptability of *Microcystis* through enhancing cell density, cell buoyancy via gas vesicle formation, EPSs and MCs secretion, nutrition, and energy metabolism, which ultimately causes cell aggregation and colony formation of *Microcystis* to strengthen its survival and competitive advantages. Notably, differences in molecular structures of exogenous AHLs and/or exogenous AHLs’ structural analogs secreted by other microorganisms cause distinct regulatory effects on *Microcystis* physiology and behavior, especially those secreted by some specific bacteria may pose inhibitory or quenching effect on *Microcystis* QS. This suggests that applying a QS inhibitor to interfere with the QS-signaling pathway could be a promising strategy for MCBs control.

However, several deficiencies in the field of *Microcystis* QS and QQ still exist. To further explore genetic mechanisms at the molecular level, as well as species distribution and the complex roles of QS signals under the influence of environmental factors, is imperatively desirable. Overall, by encompassing research progress, we provide an updated comprehensive review of *Microcystis* QS and propose new insights on future research directions to advance the understanding of underlying mechanisms.

## Figures and Tables

**Table 1:** AHLs homologs as QS signaling molecules extracted from *Microcystis.*

Microcystis Strains	AHLs Homologs	Culture Media	Structure of AHLs Homologs	References
*M. aeruginosa* FACHB-905	AHLs	BG-11	Unknown	[28]
*M. aeruginosa* PCC-7820	AHLs	BG-11	Unknown	[41]
*M. aeruginosa* HB-836	3-OXO-C5-HSL	BG-11	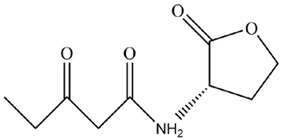	[42]
	C6-HSL	BG-11	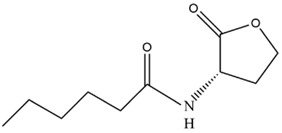	
	3-OXO-C7-HSL	BG-11	Unknown	
	3-OH-C4-HSL	BG-11	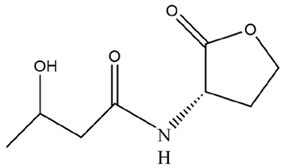	
*M. aeruginosa* FACHB-905	AHLs(C13H19O8N)	BG-11	Unknown	[43]
*M. aeruginosa* PCC7806	C3-HSL	BG-11	Unknown	[44]
	C4-HSL	BG-11	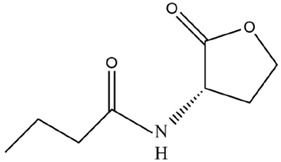	
*M. aeruginosa* PCC-7820	C8-HSLs	Allen-BG11	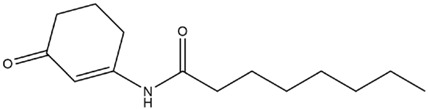	[45]

**Table 2:** Regulatory effects of exogenous AHLs addition on various aspects of *Microcystis.*

*Microcystis* Strains	Concentration	AHLs	Cell Growth	Cellular Morphology	Colony Formation	Chlorophyll a content	Photosynthesis	Superoxide Free Radical Content	Phycocyanin content	Intracellular Polysaccharide content	MCs content	EPSs content	References
*M. aeruginosa* HB909	0.2 μmol/L	3-OH-C4-HSL	√	√			√				×		[29]
		C4-HSL	√	√			√				×	√	
		C8-HSL	×	√			-				√	√	
*M. aeruginosa* PCC-7820	0.02 μmol/L	AHLs (Unknown type)		√	√								[41]
*M. aeruginosa* FACHB905			√			√	√				√	√	[43]
*M. aeruginosa* FACHB905	50 μmol/L	C4H7NO2·HBr	√										[64]
*M. aeruginosa* FACHB905	5 ng/L	C6-HSL				×							[65]
	10 ng/L					√							
	50 ng/L					×							
	500 ng/L					×							
	1000 ng/L					×							
*M. aeruginosa* FACHB905	5 μmol/L	C4H7NO2·HBr	-										[66]
	10 μmol/L		×										
	20 μmol/L		×										
	40 μmol/L		×										
	50 μmol/L					×	×	√	×	√			
	60 μmol/L		×										
	80 μmol/L		×										
	100 μmol/L		×										
*M. aeruginosa*	0.004 μmol/L	3-oxo-C10-HSL	×		-								[67]
		C6-HSL	√		√								
		C10-HSL	×		-								
		C7-HSL	√		√								
		C12-HSL	√		√								
		C8-HSL	×		√								
		3-oxo-C8-HSL	√		√								
		C4-HSL	×		√								
*M. aeruginosa* FACHB905	10 μmol/L	C4H7NO2·HBr	-					√	×	√			[73]
	20 μmol/L		-					√	×	√			
	40 μmol/L		×					√	×	√			
	60 μmol/L		×					√	×	√			
	80 μmol/L		×					√	×	√			

√: positive effect was observed; ×: negative effect was observed; -: no valid or stable effect was observed.

## Data Availability

No new data were created or analyzed in this study. Data sharing is not applicable to this article.
